# A Dose‐Tailored Anti‐Plasma Cell Regimen Lowers the Mortality of Late‐Stage Cardiac Amyloidosis

**DOI:** 10.1002/mco2.70219

**Published:** 2025-07-02

**Authors:** Yun Ti, Huaitao Yu, Ying Wang, Mei Ni, Tongtao Liu, Luqun Wang, Cheng Zhang, Peili Bu, Yun Zhang

**Affiliations:** ^1^ State Key Laboratory for Innovation and Transformation of Luobing Theory, Key Laboratory of Cardiovascular Remodeling and Function Research of MOE, NHC, CAMS and Shandong Province, Department of Cardiology Qilu Hospital of Shandong University Jinan China; ^2^ Department of Hematology Qilu Hospital of Shandong University Jinan China

**Keywords:** cardiac amyloidosis, cardiac response, dose‐tailored anti‐plasma cell regimen, heart failure, hematologic response

## Abstract

A major obstacle to using current guideline‐recommended chemotherapy in patients with advanced light‐chain cardiac amyloidosis (LCCA) is their intolerance to standard drug dosages. The study aimed to assess the efficacy and safety of the dose‐tailored BD and DBD regimen proposed by our team for patients with LCCA at Mayo Stage III. A total of 119 patients who met the inclusion and exclusion criteria for cardiac amyloidosis were recruited and divided into three groups: group A, group B, and group C who received supportive therapy, dose‐tailored BD regimen, and dose‐tailored DBD regimen, respectively. Survival rate and time, hematologic and cardiac response, and adverse events were evaluated during a median follow‐up of 30.2 months. No significant differences in baseline characteristics were found among the three groups. Group B and C showed increased survival rates and time compared to group A. Group C showed improved hematologic and cardiac responses relative to group B. Additionally, group C showed fewer adverse events related to chemotherapy compared to group B. Both dose‐tailored BD and DBD regimens increased survival rates and time in advanced LCCA patients, with the dose‐tailored DBD regimen demonstrating superior efficacy and safety. Further randomized clinical trials are needed to confirm these preliminary findings.

## Graphical abstract

The present study was designed to assess the efficacy and safety of a dose‐tailored BD regimen and a dose‐tailored DBD regimen in patients with LCCA at Mayo Stage III. Both the two regimens markedly increased survival rate and time in those patients and the dose‐tailored DBD regimen was superior to the dose‐tailored BD regimen in both efficacy and safety. BD, bortezomib and dexamethasone; DBD, daratumumab, bortezomib, and dexamethasone; LCCA, light‐chain cardiac amyloidosis.

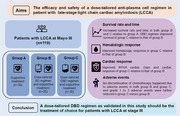

## 1 Introduction

Light‐chain cardiac amyloidosis (LCCA) is caused by progressive myocardial infiltration of insoluble amyloid fibrils that are transformed from abnormal immunoglobulin produced by clonal proliferative plasma cells. Heart is one of the organs most commonly affected by amyloidosis manifested with heart failure and cardiac arrhythmias [1]. LCCA carries the worst prognosis for patients with any type of systemic amyloidosis, with a median survival of only 3 months in high‐risk patients [2].

As both LCCA and multiple myeloma are caused by clonal proliferative disease of plasma cells, and may coexist in many patients, therapeutic strategies for multiple myeloma are commonly used to treat patients with LCCA [3, 4]. However, 27% of the Mayo Stage III patients with LCCA treated with chemotherapy still died within 3 months of diagnosis due to organ failure caused by disease progression and treatment toxicity, with an estimated 2‐year survival rate of only 29% [5]. To improve the efficacy of chemotherapy in patients with systemic light‐chain systemic amyloidosis, British Committee for Standards in Haematology (BCSH) in 2015, Mayo Clinic in 2015, EHA‐ISA working group guidelines in 2022, and National Comprehensive Cancer Network (NCCN) in 2023 [6, 7, 8, 9] issued a number of guidelines or consensuses for the diagnosis and treatment of systemic light‐chain amyloidosis. Recently, our group has issued a new expert consensus specifically for the diagnosis and treatment of LCCA [10].

In all these scientific documents, three chemotherapy regimens were recommended: (1) BD regimen involving bortezomib (1.3 mg/m^2^) and dexamethasone (20 mg); (2) BCD regimen including bortezomib (1.3 mg/m^2^), cyclophosphamide (300 mg/m^2^), and dexamethasone (20 mg); and (3) DBCD regimen comprising daratumumab (16 mg/kg), bortezomib (1.3 mg/m^2^), cyclophosphamide (300 mg/m^2^), and dexamethasone (20 mg). However, the incidence of heart failure induced by the toxicity of bortezomib (1.3 mg/m^2^) and dexamethasone (40 mg) was found to be 15% and 13%, respectively, in patients with relapsed multiple myeloma [11]. In addition, treatment with bortezomib (1.3 mg/m^2^) resulted in a higher incidence of at least Grade 3 gastrointestinal toxicities of any grade (82% with bortezomib vs. 75% without bortezomib) [12]. In our experience, most patients with Mayo Stage III LCCA are too fragile to tolerate bortezomib at the dose of 1.3 mg/m^2^, dexamethasone at the dose of 20 mg or cyclophosphamide at even a very low dose. Moreover, a Phase 2 study evaluated daratumumab (16 mg/kg) monotherapy in 40 patients with Mayo Stage III LCCA and found at least Grade 3 treatment‐related adverse events occurred in 40.0% of patients [13]. SIRIUS trial also showed a higher incidence of adverse events in the daratumumab (16 mg/kg) group [14]. Consistently, the US FDA Adverse Events Reporting System (FAERS) database reported a high incidence of cardiotoxicity induced by daratumumab (16 mg/kg) [15]. In our experience, a high incidence of gastrointestinal toxicities and severe infection were frequently observed in patients with Mayo Stage III LCCA treated with daratumumab (16 mg/kg).

Taken together, a major obstacle to applications of current guideline‐recommended chemotherapy in patients with advanced LCCA is the intolerance of these patients to standard dose of drugs. To overcome this difficulty, we have proposed two dose‐tailored chemotherapy regiments: (1) dose‐tailored BD regimen including bortezomib (1.0 mg/m^2^) and dexamethasone (10 mg); and (2) dose‐tailored DBD regimen including daratumumab (12 mg/kg), bortezomib (1.0 mg/m^2^), and dexamethasone (10 mg). In an attempt to assess the efficacy and safety of the two regiments, we have compared the therapeutic effects, adverse events and clinical outcomes in three groups of patients with LCCA at Mayo Stage III in the present study: group A patients who received only supportive treatment without chemotherapy, group B patients who received dose‐tailored BD regimen and supportive treatment, and group C patients who received dose‐tailored DBD regimen and supportive treatment.

## 2 Results

### 2.1 Baseline Characteristics

Of 119 recruited patients, 74 (62.2%) were men and the mean age was 60.4 ± 9.6 years. The time duration from symptom onset to diagnosis was on the average 8 months in group A and 6 months in group B and group C. Most patients were classified as Mayo Stage IIIa (70.6%) and New York Heart Association (NYHA) cardiac function class III (51.3%). The majority of patients (83.2%) exhibited typical electrocardiogram (ECG) features of LCCA, that is, a low voltage in the limb leads and Q‐waves and poor R‐wave increments in leads V1–V3. Liver and kidney were involved by amyloidosis in 17.6% and 40.3% patients, respectively. The serum levels of difference between involved and uninvolved serum immunoglobulin‐free light‐chain levels (dFLC), N‐terminal‐pro B type natriuretic peptide (NT‐proBNP), and cardiac troponin I (cTnI) were significantly increased. Both interventricular septum thickness at end diastole (IVSd) and left ventricular posterior wall thickness at end diastole (LVPWd) were thickened. Although the mean left ventricular ejection fraction (LVEF) was in the normal range, the mean left ventricular global longitudinal strain (LVGLS) was markedly decreased. A small proportion of patients had combined diabetes, hypertension, coronary artery disease and myeloma. Positive Congo red staining was detected from biopsy samples of myocardium in three patients (2.5%), kidney in 23 patients (19.3%), liver in one patient (0.9%), abdominal subcutaneous adipose tissue in 22 patients (18.5%), neuromuscular tissue in 25 patients (21%), tongue in three patients (2.5%), bladder tissue in one patient (0.9%), gastrointestinal tract in four patients (3.4%), and bone marrow biopsy in 37 patients (31%). Most patients received beta‐blocker and diuretics therapies. Baseline characteristics of the 119 patients were shown in Table 1.

**TABLE 1:** Baseline characteristics of all recruited patients with light‐chain cardiac amyloidosis (LCCA) at Mayo Stage III.

Characteristics	Overall
Age (years), mean ± SD	60.4 ± 9.6
Male, *n* (%)	74 (62.2%)
Time from symptom onset to diagnosis(months), median (IQR)	6 (3, 12)
Stage, *n* (%)	
IIIa	84 (70.6%)
IIIb	35 (29.4%)
NYHA, *n* (%)	
II	29 (24.4%)
III	61 (51.3%)
IV	29 (24.4%)
Typical ECG manifestations, *n* (%)	99 (83.2%)
Liver involvement, *n* (%)	21 (17.6%)
Renal involvement, *n* (%)	48 (40.3%)
Light‐chain isotype, *n* (%)	
*κ*	50 (42%)
*λ*	69 (58%)
SBP (mm Hg), median (IQR)	107 (98, 126)
DBP (mm Hg), median (IQR)	69 (61, 78)
dFLC (mg/L), median (IQR)	267.89 (115.13, 539.26)
NT‐proBNP (ng/L), median (IQR)	5496.00 (2490.00, 10,550.00)
cTnI (µg/L), median (IQR)	0.06 (0.03, 0.13)
Cr (µmol/L), median (IQR)	83.00 (67.00, 107.50)
eGFR (mL/min/1.73 m^2^), median (IQR)	74.60 (53.79, 93.59)
AKP (U/L), median (IQR)	86.00 (62.00, 127.00)
Alb (g/L), median (IQR) 24 h urine protein (g/24 h), median (IQR)	37.30 (30.65, 40.30) 0.24 (0.11, 1.88)
WBC (× 10^9^/L), median (IQR)	6.27 (4.9675, 7.49)
HGB (g/L), median (IQR)	122.50 (110.25, 137.00)
IVS (mm), median (IQR)	14 (13, 16)
LVPW (mm), median (IQR)	13 (12, 15)
LVEF (%), mean ± SD	50.80 ± 11.11
LVGLS (%), mean ± SD	−11.94 ± 3.99
Diabetes, *n* (%)	14 (11.8%)
Hypertension, *n* (%)	35 (29.4%)
CAD, *n* (%)	31 (26.1%)
Myeloma, *n* (%)	23 (19.3%)
Proportion of abnormal plasma cells (%), median (IQR)	3.82 (2.00, 9.18)
ACEI, *n* (%)	6 (5%)
ARB, *n* (%)	10 (8.4%)
ARNI, *n* (%)	10 (8.5%)
Beta‐blockers, *n* (%)	57 (47.9%)
SGLT‐2 inhibitors, *n* (%)	11 (9.2%)
Diuretics, *n* (%)	93 (78.2%)
Loop diuretics, *n* (%)	90 (75.6%)
Aldosterone antagonist, *n* (%)	88 (73.9%)
Tolvaptan, *n* (%)	14 (11.8%)

Abbreviations: ACEI, angiotensin‐converting enzyme inhibitor; AKP, alkaline phosphatase; Alb, albumin; ARB, angiotensin‐II receptor blocker; ARNI, angiotensin receptor neprilysin inhibitor; CAD, coronary artery disease; Cr, creatinine; cTnI, cardiac troponin I; DBP, diastolic blood pressure; dFLC, the difference between involved and uninvolved serum immunoglobulin‐free light‐chain levels; ECG, electrocardiogram; eGFR, estimated glomerular filtration rate; IVS, interventricular septum; LVEF, left ventricular ejection fraction; LVGLS, left ventricular global longitudinal strain; LVPW, left ventricular posterior wall; NT‐proBNP, N‐terminal‐pro B type natriuretic peptide; NYHA, New York Heart Association; SBP, systolic blood pressure; SGLT‐2, sodium‐glucose cotransporter‐2; Stage, Mayo 2004 cardiac AL staging system.

Baseline clinical characteristics of patients in three groups are shown in Table 2. There were 59, 25, and 35 patients in group A, group B, and group C, respectively. No significant difference was observed in age, gender, systolic and diastolic blood pressures, cardiac function classification, Mayo stage classification, time duration from symptom onset to diagnosis, typical ECG features, light chain (*κ* or *λ*) involved, liver and renal amyloidosis, and combined coronary artery disease, hypertension, diabetes, and myeloma among the three groups of patients. In addition, there was no significant difference in medications administered among the three groups of patients except for beta‐blockers (*p* = 0.006) and SGLT‐2 inhibitors (*p* = 0.003) which were more commonly used in group A and group C, respectively, than the other two groups.

**TABLE 2:** Comparison of baseline characteristics among three groups of patients.

Characteristics	Group A (*n* = 59)	Group B (*n* = 25)	Group C (*n* = 35)	*p* value
Age (years), mean ± SD	60.56 ± 10.89	58.56 ± 8.71	61.34 ± 7.82	0.449
Male, *n* (%)	35 (59.3%)	15 (60.0%)	24 (68.6%)	0.649
Time from symptom onset to diagnosis (months), median (IQR)	8 (4.0, 12.0)	6 (3.0, 12.0)	6 (1.5, 12.0)	0.445
Stage, *n* (%)				0.201
IIIa	41 (69.5%)	22 (62.9%)	21 (84.0%)	
IIIb	18 (30.5%)	13 (37.1%)	4 (16%)	
NYHA, *n* (%)				0.661
II	11 (18.6%)	10 (28.6%)	8 (32.0%)	
III	33 (55.9%)	16 (45.7%)	12 (48.0%)	
IV	15 (25.4%)	9 (25.7%)	5 (20.0%)	
Typical ECG manifestations, *n* (%)	50 (84.7%)	21 (84.0%)	28 (80.0%)	0.832
Liver involvement, *n* (%)	11 (18.6%)	4 (16.0%)	6 (17.1%)	0.954
Renal involvement, *n* (%)	20 (33.9%)	11 (44.0%)	17 (48.6%)	0.343
Light‐chain isotype, *n* (%)				0.062
*κ*	28 (47.5%)	13 (52.0%)	9 (25.7%)	
*λ*	31 (52.5%)	12 (48.0%)	26 (74.3%)	
SBP (mm Hg), median (IQR)	106 (96.5, 124.0)	110 (99.0, 124.0)	109 (99.0, 129.0)	0.747
DBP (mm Hg), median (IQR)	67 (60.0, 76.0)	73 (63.0, 79.0)	70 (63.0, 75.5)	0.421
Diabetes, *n* (%)	9 (15.3%)	2 (8.0%)	3 (8.6%)	0.502
Hypertension, *n* (%)	17 (28.8%)	6 (24.0%)	12 (34.3%)	0.683
CAD, *n* (%)	18 (30.5%)	5 (20.0%)	8 (22.9%)	0.530
Myeloma, *n* (%)	7 (11.9%)	8 (32.0%)	8 (22.9%)	0.086
Proportion of abnormal plasma cells (%), median (IQR)	3.00 (2.01, 9.25)	4.64 (2.53, 10.00)	3.55 (1.56, 5.75)	0.429
ACEI, *n* (%)	5 (8.5%)	0 (0.0%)	1 (2.9%)	0.209
ARB, *n* (%)	7 (11.9%)	0 (0.0%)	3 (8.6%)	0.201
ARNI, *n* (%)	2 (3.4%)	3 (12.0%)	5 (14.7%)	0.131
Beta‐blockers, *n* (%)	37 (62.7%)	8 (32.0%)	12 (34.3%)	0.006
SGLT‐2 inhibitors, *n* (%)	1 (1.7%)	2 (8.0%)	8 (22.9%)	0.003
Diuretics, *n* (%)	48 (81.4%)	20 (80.0%)	25 (71.4%)	0.514
Loop diuretics, *n* (%)	38 (64.4%)	23 (92%)	29 (82.9%)	0.013
Aldosterone antagonist, *n* (%)	41 (69.5%)	19 (76%)	28 (80%)	0.515
Tolvaptan, *n* (%)	3 (5.1%)	3 (12%)	8 (22.9%)	0.035

Abbreviations: ACEI, angiotensin‐converting enzyme inhibitor; ARB, angiotensin‐II receptor blocker; ARNI, angiotensin receptor neprilysin inhibitor; CAD, coronary artery disease; DBP, diastolic blood pressure; ECG, electrocardiogram; NYHA, New York Heart Association; SBP, systolic blood pressure; SGLT‐2, sodium‐glucose cotransporter‐2.; Stage, Mayo 2004 cardiac AL staging system.

Baseline laboratory characteristics of patients in three groups were shown in Table 3. There were no differences among the three groups of patients in terms of the serum levels of NT‐proBNP, cTnI, dFLC, Cr, 24 h urine protein, AKP and Alb, white blood cells (WBC), HGB, eGFR, as well as echocardiographic measurements of IVS, LVPW, LVEF, and LVGLS.

**TABLE 3:** Comparison of laboratory and echocardiographic characteristics of patients among three groups of patients.

Characteristics	Group A (*n* = 59)	Group B (*n* = 25)	Group C (*n* = 35)	*p* value
NT‐proBNP (ng/L), median (IQR)	5924.00 (3208.50, 11,020.00)	5655.00 (1955.00, 11,200.00)	3813.00 (2109.00, 8113.00)	0.281
cTnI (µg/L), median (IQR)	0.07 (0.03, 0.15)	0.05 (0.03, 0.09)	0.04 (0.02, 0.11)	0.177
dFLC (mg/L), median (IQR)	184.11 (126.76, 634.29)	398.30 (187.67, 1143.00)	204.16 (98.31, 360.10)	0.089
Cr (µmol/L), median (IQR)	80.00 (66.50, 105.00)	86.00 (73.00, 119.00)	80.00 (67.00, 98.50)	0.461
eGFR (mL/min/1.73 m^2^), mean ± SD	76.20 ± 34.27	67.23 ± 36.73	76.12 ± 24.32	0.470
24 h urine protein (g/24 h), median (IQR)	0.18 (0.11, 1.08)	0.34 (0.14, 1.45)	0.48 (0.12, 1.99)	0.334
AKP (U/L), median (IQR)	79.00 (62.50, 149.50)	97.00 (69.00, 108.00)	93 (62.00, 127.50)	0.880
Alb (g/L), median (IQR)	36.90 (30.65, 40.00)	39.10 (31.60, 42.40)	37.10 (30.35, 39.90)	0.586
WBC (× 10^9^/L), median (IQR)	6.35 (5.17, 7.75)	5.87 (4.83, 6.86)	6.34 (5.28, 7.45)	0.590
HGB (g/L), median (IQR)	119.00 (104.00, 135.00)	124.00 (116.00, 144.00)	125.50 (110.50, 137.00)	0.281
IVS (mm), median (IQR)	15.00 (14.00, 17.00)	14.00 (13.00, 16.00)	14.00 (13.00, 16.00)	0.285
LVPW (mm), median (IQR)	14.00 (12.00, 15.00)	13.00 (12.75, 15.00)	13.00 (11.00, 14.00)	0.129
LVEF (%), mean ± SD	48.73 ± 12.38	52.28 ± 11.14	53.23 ± 8.01	0.124
LVGLS (%), median (IQR)	−13.70 (−15.10, −10.60)	−10.10 (−13.23, −8.03)	−14.50 (−15.60, −7.80)	0.107

Abbreviations: AKP, alkaline phosphatase; Alb, albumin; Cr, creatinine; cTnI, cardiac troponin I; eGFR, estimated glomerular filtration rate; HGB, hemoglobin; IVS, interventricular septum; LVEF, left ventricular ejection fraction; LVGLS, left ventricular global longitudinal strain; LVPW, left ventricular posterior wall; NT‐proBNP, N‐terminal probrain natriuretic peptide; WBC, white blood cells.

### 2.2 Survival Rate and Time

**FIGURE 1:**
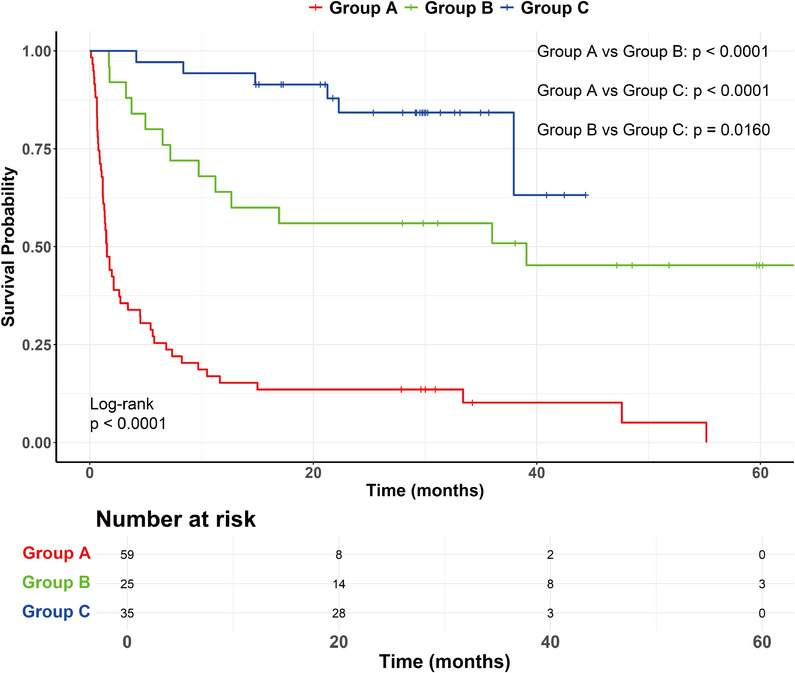
Comparison of survival rate among three groups of patients. The red, green, and blue curve represents group A, group B, and group C, respectively. *p* value: Kaplan–Meier method (log‐rank test). Statistical significance: *p* < 0.05. *n* = 59 for group A, *n* = 25 for group B, *n* = 35 for group C.

As shown in Figure 1, the median follow‐up time was 30.2 months for three groups of patients. The estimated survival rate at 3, 6, 12, and 24 months was 35.6%, 25.4%, 15.3%, and 13.6% in group A, 92%, 80%, 64%, and 55.7% in group B, and 100%, 97%, 93.7%, and 86.2% in group C. There was a significant difference in survival rate among the three groups of patients (*p* < 0.0001). Compared with group A, the survival rate of group B [cHR (hazard ratio) = 0.219 (0.117–0.410), *p* < 0.001] and group C [cHR = 0.075 (0.032–0.177), *p* < 0.001] was markedly increased (Table 4). In addition, the survival rate of group C was significantly higher than that of group B (*p* = 0.016). The median survival time of patients in group A was 1.5 months and 39.1 months in group B, respectively. Because the majority of patients in group C was still alive by the end of patient recruitment, the median survival time could not be determined in this group. Figure 1 showed the variation of survival probability over time for three groups of patients.

**TABLE 4:** Univariate and multivariate Cox‐regression analysis of predictors of death in the whole cohort.

Variables	HR (95% CI)	*p* value
**Univariate Cox‐regression analysis**		
Treatment		
Group A	1.000	
Group B	0.219 (0.117–0.410)	< 0.001
Group C	0.075 (0.032–0.177)	< 0.001
Stage		
IIIa	1.000	
IIIb	1.752 (1.074–2.858)	0.025
Typical ECG manifestations		
Yes	1.000	
No	0.415 (0.198–0.867)	0.019
SGLT‐2 inhibitors		
No	1.000	
Yes	0.303 (0.095–0.964)	0.043
Sex		
Men	1.000	
Women	1.211 (0.753–1.946)	0.429
Age, years		
< 65	1.000	
≥ 65	1.019 (0.630–1.648)	0.940
**Multivariate Cox‐regression analysis**		
Treatment		
Group A	1.000	
Group B	0.155 (0.078–0.308)	< 0.001
Group C	0.035 (0.014–0.091)	< 0.001
Stage		
IIIa	1.000	
IIIb	3.447 (2.001–5.938)	< 0.001
Typical ECG manifestations		
Yes	1.000	
No	0.244 (0.110–0.541)	< 0.001
SGLT‐2 inhibitors		
No	1.000	
Yes	1.307 (0.380–4.492)	0.981

Abbreviations: 95% CI, 95% confidence interval; ECG, electrocardiogram; HR, hazard ratio; SGLT‐2, sodium‐glucose cotransporter‐2.

### 2.3 Predictors of Death

Univariate Cox‐regression analysis was performed to identify the risk factors of death, which showed that group B, group C, and use of SGLT‐2 inhibitors were three significant predictors of a lower risk of death whereas Stage III b and ECG features were two significant predictors of a higher risk of death. Based on these predictors, multivariate Cox‐regression analysis was conducted to screen independent predictors of outcome, which identified that group B and group C were two independent predictors of a lower risk of death while Stage III b and ECG features were two independent predictors of a higher risk of death. By comparison, the value of HR for group C as a predictor [0.035 (0.014–0.091)] was much smaller than that for group B [0.155 (0.078–0.308)], suggesting that group C was a more powerful predictor of a lower risk of death than group B. In addition, group B and group C remained independent predictors of a lower risk of death even after controlling for Stage IIIb, ECG features, and use of SGLT‐2 inhibitors (Table 4).

### 2.4 Hematologic Response to Chemotherapy

**FIGURE 2:**
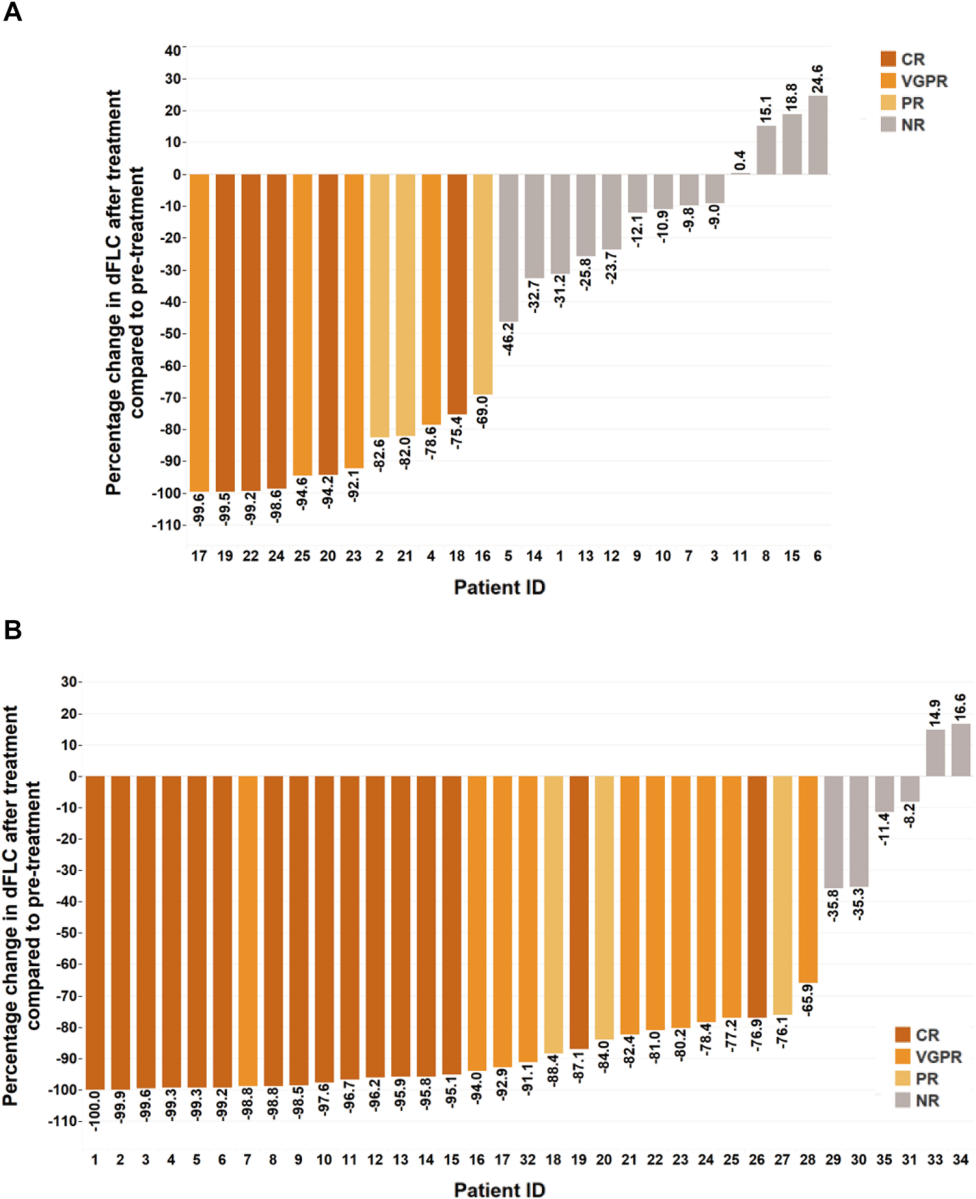
Percentage change of dFLC 3 months after the initiation of chemotherapy in group B and group C. Waterfall plot showing percentage change of dFLC 3 months after the initiation of chemotherapy in each patient of group B (A) and group C (B). CR, complete response; dFLC, difference between involved and uninvolved serum immunoglobulin‐free light‐chain levels; PR, partial response; VGPR, very good partial response.

Hematologic response was evaluated 3 months after the initiation of chemotherapy. The median duration of chemotherapy was 5.5 (3.25–8, interquartile) cycles in group B and 2.5 (1.5–3.65, interquartile) cycles in group C. Chemotherapy was terminated when the patient died, and discontinued if the patient manifested with severe side effects or complete remission (CR), in whom, however, clinical follow‐up was continued. For hematologic response to chemotherapy in group B, 12 patients achieved overall response rate (ORR, 48%) including five patients with complete response (CR), four patients with very good partial response (VGPR), and three patients with partial response (PR). For hematologic response to chemotherapy in group C, 29 patients achieved ORR (82.9%) including 16 patients with CR, 10 patients with VGPR, and three patients with PR. The values of ORR (*p* = 0.004) and the sum of CR and VGPR (*p* = 0.003) were significantly higher in group C than group B. The hematologic response and the percentage change of dFLC after chemotherapy in both groups were shown in Table 5 and Figure 2.

**TABLE 5:** Comparison of hematologic and cardiac responses to chemotherapy between group B and group C.

Characteristics	Group B (*n* = 25)	Group C (*n* = 35)	*p* value
**Hematologic response, *n* (%)**			
ORR	12 (48.0%)	29 (82.9%)	0.004
≥ VGPR	9 (36.0%)	26 (74.3%)	0.003
CR	5 (20.0%)	16 (45.7%)	
VGPR	4 (16.0%)	10 (28.6%)	
PR	3 (12.0%)	3 (8.6%)	
NR	13 (52.0%)	6 (17.1%)	
**Cardiac response, *n* (%)**			
ORR	9 (36.0%)	20 (57.1%)	0.106
≥ VGPR	4 (16.0%)	12 (34.3%)	0.114
CR	1 (4.0%)	2 (5.7%)	
VGPR	3 (12.0%)	10 (28.6%)	
PR	5 (20.0%)	8 (22.8%)	
NR	16 (64.0%)	15 (42.9%)	

Abbreviations: CR, complete remission; NR, no response; ORR, overall response rate; PR, partial response; VGPR, very good partial response.

### 2.5 Cardiac Response to Chemotherapy

**FIGURE 3:**
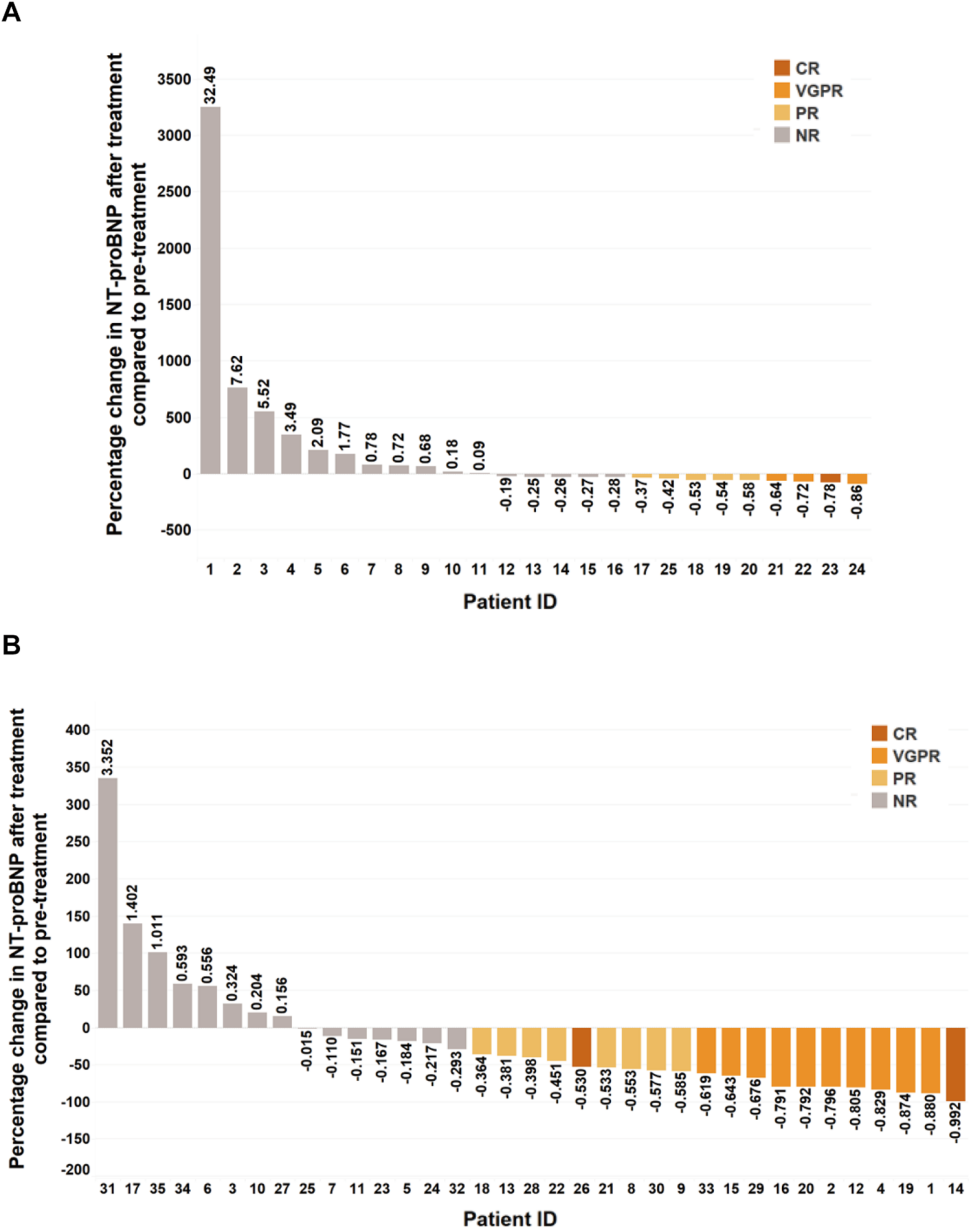
Percentage change of NT‐proBNP 6 months after the initiation of chemotherapy in group B and group C. Waterfall plot showing percent change of NT‐proBNP 6 months after the initiation of chemotherapy in each patient of group B (A) and group C (B). CR, complete response; NT‐proBNP, N‐terminal‐pro B type natriuretic peptide; PR, partial response; VGPR, very good partial response.

Cardiac response was evaluated 3 and 6 months, respectively, after the initiation of chemotherapy. For cardiac response to 3 months of chemotherapy in group B, six patients achieved ORR (24%) including two patients with VGPR and four patients with PR. For cardiac response to 3 months of chemotherapy in group C, 13 patients achieved ORR (37.1%) including three patients with VGPR and 10 patients with PR (Supporting Information Table 1). For cardiac response to 6 months of chemotherapy in group B, nine patients achieved ORR (36%) including one patient with CR, three patients with VGPR, and five patients with PR. For cardiac response to 6 months of chemotherapy in group C, 20 patients achieved ORR (57.1%) including two patients with CR, 10 patients with VGPR and eight patients with PR (Table 5). Although more patients achieved ORR in group C than in group B, there was no significant deference in ORR and the sum of CR and VGPR between the two groups. The cardiac response to chemotherapy in both groups is shown in Table 5. The individual percentage change of NT‐proBNP after 3 months of chemotherapy in both groups were shown in Supporting Information Figure 1. The individual percentage change of NT‐proBNP after 6 months of chemotherapy in both groups was shown in Figure 3.

**FIGURE 4:**
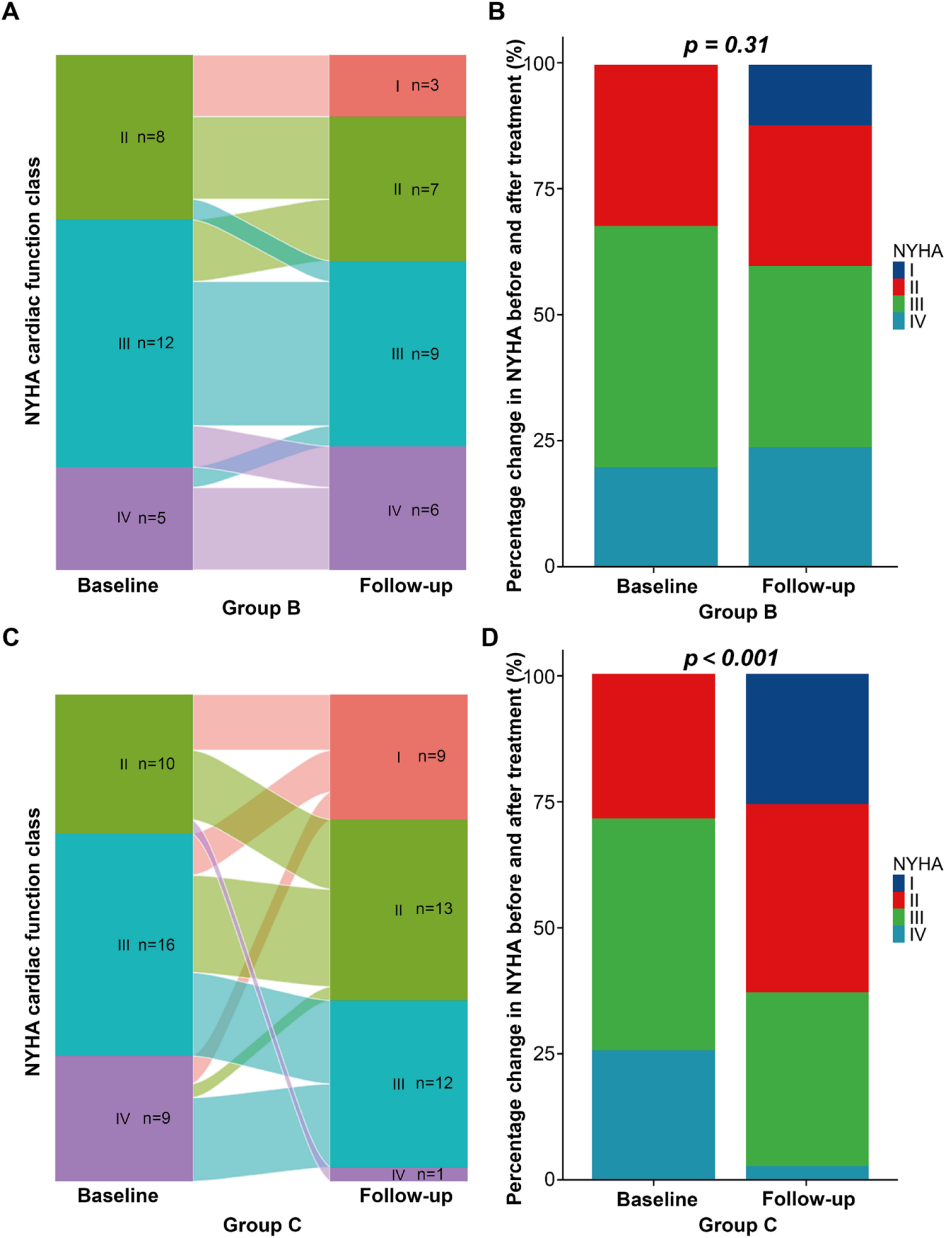
Changes in NYHA cardiac function class 6 months after the initiation of chemotherapy in group B and group C. Sankey diagram of changes in NYHA cardiac function class in group B (A) and group C (C). Stacked proportional histograms of changes in NYHA cardiac function class in group B (B) and group C (D). *p* value: Continuous corrective chi‐square test (Yates' correction) for stacked proportional histograms. Statistical significance: *p* < 0.05. *n* = 25 for group B, *n* = 35 for group C. NYHA, New York Heart Association.

The categorical changes of NYHA cardiac function class after chemotherapy in group B and group C were shown in Figure 4. Improvement of one NYHA class was achieved in seven patients (28.0%) of group B and in 17 patients (48.6%) of group C after chemotherapy. Improvement of two and three NYHA classes was achieved in five patients (14.3%) and two patients (5.7%), respectively, in group C after chemotherapy. There was a significant improvement of NYHA class in group C after chemotherapy (*p* < 0.001) while such an improvement was not significant in group B.

**FIGURE 5:**
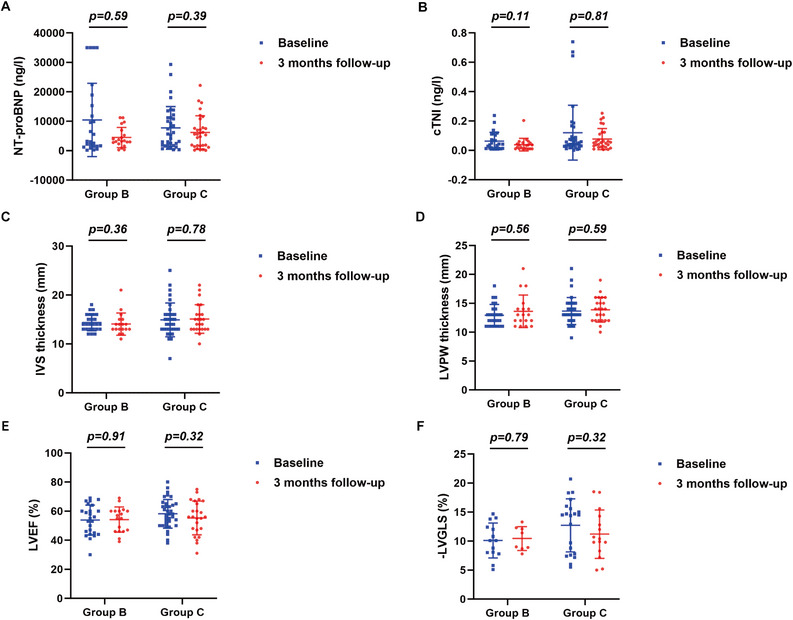
Changes in NT‐proBNP, cTnI, and echocardiographic measurements 3 months after the initiation of chemotherapy in group B and group C. Scatter plots representing changes in NT‐proBNP (A), cTnI (B), IVS thickness (C), LVPW thickness (D), LVEF (E), LVGLS (F) 3 months after the initiation of chemotherapy in group B and group C. *p* value: Mann–Whitney *U*‐test (Wilcoxon rank sum test) for NT‐proBNP, cTnI, IVS thickness, and LVPW thickness; independent samples *T*‐test for LVEF and LVGLS. Statistical significance: *p* < 0.05. *n* = 20–35 for NT‐proBNP and cTnI, *n* = 18–35 for IVS thickness and LVPW thickness, *n* = 8–35 for LVEF and LVGLS. cTNI, cardiac troponin I; IVS, interventricular septal; LVEF, left ventricular ejection fraction; LVGLS, left ventricular global longitudinal strain; LVPW, left ventricular posterior wall; NT‐proBNP, N‐terminal‐pro B type natriuretic peptide.

**FIGURE 6:**
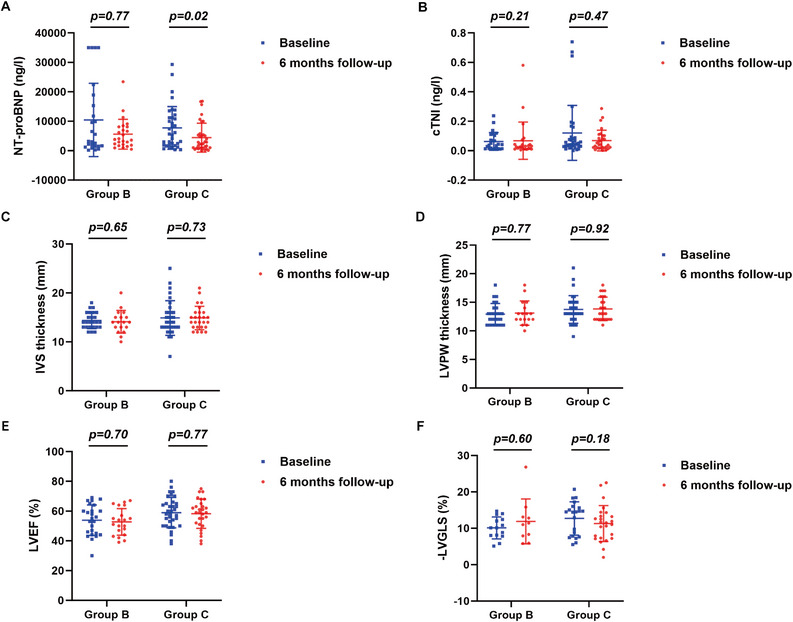
Changes in NT‐proBNP, cTnI, and echocardiographic measurements 6 months after the initiation of chemotherapy in group B and group C. Scatter plots representing changes in NT‐proBNP (A), cTnI (B), IVS thickness (C), LVPW thickness (D), LVEF (E), LVGLS (F) 6 months after the initiation of chemotherapy in group B and group C. *p* value: Mann–Whitney *U*‐test (Wilcoxon rank sum test) for NT‐proBNP, cTnI, IVS thickness, LVPW thickness, and LVGLS; Independent samples *T*‐test for LVEF. Statistical significance: *p* < 0.05. *N* = 24–35 for NT‐proBNP and cTnI, *N* = 17–33 for IVS thickness and LVPW thickness, and *N* = 10–35 for LVEF and LVGLS. NT‐proBNP, N‐terminal‐pro B type natriuretic peptide; cTNI, cardiac troponin I; IVS, interventricular septal; LVPW, left ventricular posterior wall; LVEF, left ventricular ejection fraction; LVGLS, left ventricular global longitudinal strain.

Serum levels of NT‐proBNP and cTnI, and echocardiographic measurements before and 3, 6, and 12 months after chemotherapy in group B and group C were shown in Figures 5, 6 and Supporting Information Figure 2. Compared with baseline measurements, no significant changes in serum levels of NT‐proBNP and cTnI, and echocardiographic values were observed 3 months after chemotherapy in both group B and group C (Figure 5). However, serum levels of NT‐proBNP showed a significant decline 6 months (*p* = 0.020, Figure 6A) and 12 months (*p* = 0.030, Supporting Information Figure 2A) after chemotherapy in group C, but exhibited no significant changes 6 and 12 months after chemotherapy in group B. In contrast, in both group B and group C, serum levels of cTnI showed no noticeable changes after chemotherapy relative to baseline levels. Similarly, values of IVS and LVPW, LVEF and LVGLS showed no significant changes 6 and 12 months after chemotherapy in both group B and group C.

### 2.6 Safety of Chemotherapy

The adverse events that occurred during treatment in both groups were shown in Table 6. No Grade 4 chemotherapy‐related adverse events occurred in either group B or group C. Grade 1–3 adverse events related to chemotherapy were less common in group C than group B (68% vs. 37.1%, *p* = 0.018). More patients in group B experienced a declined lymphocyte count than group C (*p* = 0.017). There were no discontinuations of dose‐tailored anti‐plasma cell therapy due to adverse events during the entire course of treatment.

**TABLE 6:** Comparison of adverse events of anti‐plasma cell therapy between group B and group C.

	Group B				Group C				
Characteristics	All grade *n* (%)	Grade 1 n	Grade 2 *n*	Grade 3 *n*	All grade *n* (%)	Grade 1 *n*	Grade 2 *n*	Grade 3 *n*	*p* value
Total	17 (68%)				13 (37.1%)				0.018
Injection site reactions	3 (12.0%)	3	0	0	0 (0%)	0	0	0	0.133
Systemic administration reactions	5 (20.0%)	5	0	0	2 (5.7%)	2	0	0	0.197
Declined neutrophil count	1 (4.0%)	1	0	0	0 (0%)	0	0	0	0.417
Declined lymphocyte count	14 (56.0%)	10	3	1	9 (25.7%)	5	4	0	0.017
Declined platelet count	4 (16.0%)	2	2	0	1 (2.9%)	0	1	0	0.180
Elevated alanine aminotransferase	5 (20.0%)	1	2	0	1 (2.9%)	1	0	0	0.081
Elevated aspartate aminotransferase	4 (16.0%)	1	1	0	0 (0%)	0	0	0	0.054
Elevated alkaline phosphatase	2 (8.0%)	0	2	0	2 (5.7%)	1	1	0	1.000
Elevated blood bilirubin	5 (20.0%)	1	3	1	2 (5.7%)	1	1	0	0.197
Hyperuricemia	5 (20.0%)	5	0	0	9 (25.7%)	9	0	0	0.606
Urinary retention	0 (0%)	0	0	0	1 (2.9%)	0	1	0	1.000
Anemia	12 (48.0%)	7	5	0	9 (25.7%)	4	5	0	0.074
Pneumonitis	10 (40.0%)	0	10	0	10 (28.6%)	0	10	0	0.355
Urinary tract infection	2 (8.0%)	0	0	2	5 (14.3%)	0	0	5	0.734
Herpes	1 (4.0%)	0	0	1	1 (2.9%)	0	0	1	1.000
Stroke	0 (0%)	0	0	0	1 (2.9%)	0	0	1	1.000
≥ 30% decline in eGFR	7 (28.0%)	3	4	0	4 (11.4%)	1	3	0	0.195
Gastrointestinal disorders, *n* (%)	6 (24.0%)				7 (20.0%)				0.711
Abdominal distension, *n* (%)	2 (8.0%)	2	0	0	6 (17.1%)	5	0	1	0.521
Abdominal pain, *n* (%)	2 (8.0%)	1	0	1	1 (2.9%)	0	0	1	0.764
Diarrhea, *n* (%)	1 (4.0%)	1	0	0	1 (2.9%)	0	1	0	1.000
Nausea, *n* (%)	2 (8.0%)	2	0	0	1 (2.9%)	0	1	0	0.764
Vomiting, *n* (%)	2 (8.0%)	2	0	0	1 (2.9%)	0	0	1	0.764

*Note*: Adverse events of patients receiving a dose‐tailored BD regimen and a dose‐tailored DBD regimen were evaluated according to Common Terminology Criteria for Adverse Events (CTCAE) Version 5.0.

Abbreviation: eGFR, estimated glomerular filtration rate.

## 3 Discussion

There were several important findings in the present study. First, the time duration from symptom onset to diagnosis was on the average 6–8 months, leading to a majority of patients admitted with Mayo Stage III and NYHA cardiac function class III. This prolonged duration reflects a high rate of misdiagnosis of LCCA by most physicians, which shortened the treatment window for LCCA; Second, Stage III b and ECG features were independent predictors of higher risk of death in patients with LCCA; Third, both dose‐tailored BD and DBD regimens markedly increased survival rate and time in group B and group C relative to group A, indicating the vital role of chemotherapy in patients with LCCA; Fourth, DBD regimen substantially improved survival, NYHA cardiac class and hematologic and cardiac response of group C relative to that of group B; Finally, no discontinuation of chemotherapy happened due to adverse events or reactions in both group B and group C, and Grade 1–3 adverse events related to chemotherapy were further reduced in group C versus group B. These results demonstrated for the first time the high efficacy and safety of both dose‐tailored DBD and BD regimens and the superiority of dose‐tailored DBD regimen over dose‐tailored BD regimen in patients with late‐stage cardiac amyloidosis.

In clinical practice, conventional treatment such as proteasome inhibitors and immunomodulators exhibit limited efficacy in patients with LCCA. For instance, although high‐dose melphalan combined with autologous stem cell transplantation yielded favorable hematologic and organ responses in patients with LCCA, most patients with advanced cardiac amyloidosis could not tolerate these therapies, resulting in rapid disease progression or even a high mortality [16]. Unfortunately, international guidelines have not provided specific diagnostic and treatment protocols for LCCA. As a result, our group, in collaboration with Peking Union Medical College Hospital, has recently released the Chinese Consensus on the Diagnosis and Treatment of Immunoglobulin Light‐Chain Cardiac Amyloidosis [10], which advocates daratumumab as the first‐line therapy for patients with LCCA. Daratumumab, a monoclonal antibody that specifically targets the CD38 antigen, has emerged as an effective therapeutic option for AL amyloidosis [17, 18]. In a recent meta‐analysis including 997 patients with light‐chain amyloidosis, of whom 43.2% patients had cardiac involvement at Mayo Stage III, administration of daratumumab‐based therapy resulted in hematologic remission in 77% and an improved cardiac response in 41% of patients [19]. A subsequent retrospective study revealed a rapid and profound hematologic response to daratumumab in a cohort of 25 patients with severe LCCA, demonstrating an overall hematologic efficacy rate of 76% [20]. In the present study, patients receiving a dose‐tailored DBD regimen based on daratumumab (12 mg/kg) demonstrated an impressive hematologic remission rate of 82.9% and a remarkable CR rate of 45.7%, which was consistent with previous reports. Taken together, our meticulously tailored DBD regimen ensures a high therapeutic efficacy in patients with advanced cardiac amyloidosis.

In the current study, we found that the onset of cardiac remission lagged behind hematologic remission, with patients achieving hematologic remission but unchanged cardiac function 3 months after chemotherapy. These results suggested that even if anti‐plasma cell therapy successfully reduces the production of abnormal light‐chain proteins in the body, removal of amyloid that has already deposited in the heart and repair of the affected cardiac tissue is a slow process [21, 22]. Previous studies reported that patients with LCCA typically experience improvement in organ function at a median of 2.4 months after complete hematologic remission [21]. In this study, we observed cardiac remission 3 months after hematologic remission, with a decrease of NT‐proBNP levels and an improvement in NYHA cardiac function class compared to pretreatment levels. Previous studies also demonstrated that even in patients with good hematologic and cardiac responses, ventricular wall thickness improved even at a slower pace [4]. In the current study, no significant deterioration was detected in LVEF or LVGLS 6 months after chemotherapy, suggesting that a dose‐tailored DBD regimen may effectively maintain cardiac structure and halt disease progression during follow‐up. However, long‐term follow‐up is necessary to observe further improvements in cardiac remodeling.

Clinical observations demonstrate that patients with LCCA at Stage III constitute a high‐risk group, who often exhibit an impaired reserve to withstand a high‐dose anti‐plasma cell therapy while an increased sensitivity to drug toxicity. Consequently, a poor tolerance to chemotherapy contributes to the poor prognosis observed in patients with advanced stages of LCCA [5]. The objective of achieving profound and enduring responses necessitates a careful balance between therapeutic toxicity and patient safety, thus ensuring individual's survival for an adequate duration to derive benefits from anti‐plasma cell therapy and increase overall patient survival. In order to achieve effective and safe treatment, our modified regimen incorporates a downward adjustment of the drug dosage, along with meticulous cardiac monitoring to optimize the management of heart failure and arrhythmias. The incidence of Grade 3 adverse events was minimal (22.8% in group C), with no occurrence of Grade 4 adverse events. Thus, our tailored regimen is better suited for patients with advanced‐stage cardiac amyloidosis, ensuring both efficacy and patient safety.

As our study was not a randomized study, we compared our regimens with similar BD and DBD regimens reported in the literature. Using a standard BD regimen, Lamm et al. [23] reported a hematological response rate of 54%, and Kastritis et al. [17] reported a cardiac response rate of 22.2%. Our results showed a hematological response rate of 48% and a cardiac response rate of 36% using a dose‐tailored BD regimen, which was similar to previous studies. Similarly, using a standard DBD regimen, Sun et al. [19] reported a hematological response rate of 77%, a cardiac response rate of 41% and 1‐year survival rate of 76%. Our results showed a hematological response rate of 82.9%, a cardiac response rate of 57.1% and 1‐year survival rate of 94.3%. In addition, Sanchorawala et al. [24] found that 91% of their patients experienced Grade 3 or 4 adverse events whereas our results showed 22.8% of patients experienced Grade 3 adverse events and no patients experienced Grade 4 adverse events, which demonstrated a superiority of tailored DBD regimen over standard regimen.

There were several limitations to our study. First, this is a single‐center, nonrandomized, open‐labeled study. The rarity of LCCA in the Chinese population and unfamiliarity of many Chinese cardiologists to this disorder make design and performance of a multicenter and large‐scale trial extremely difficult at present. In addition, with several drugs in combination in the BD and DBD regimen groups, it is impossible to conduct a double‐blinded study. Second, the follow‐up period was relatively short. However, the marked difference in survival rate and time has been demonstrated among the three groups of patients with LCCA. Third, numerous studies have demonstrated the beneficial effects of anti‐plasma cell therapy on the prognosis of patients with LCCA. However, our study provided explicit evidence that a dose‐tailored DBD regimen was superior to a dose‐tailored BD regimen in both efficacy and safety for patients with LCCA at Stage III.

## 4 Conclusions

In conclusion, both dose‐tailored BD and DBD regimens markedly increased survival rate and time in patients with advanced LCCA, and dose‐tailored DBD regimen was superior to dose‐tailored BD regimen in both efficacy and safety. Thus, chemotherapy based on a dose‐tailored DBD regimen as validated in this study should be the treatment of choice for patients with LCCA at Mayo Stage III. Further randomized clinical trial is needed to substantiate our primary conclusion.

## 5 Methods and Materials

### 5.1 Study Design and Population

A total of 119 patients with Mayo Stage III light‐chain amyloidosis were recruited into this study. The inclusion criteria of the study population were: (1) at least 18 years of age; (2) the definite diagnosis of LCCA was established based on clinical, serum, cardiac imaging, and pathological findings; (3) the criteria of Stage III light‐chain amyloidosis were met according to the Mayo consensus 2004 [25]; (4) at least one chemotherapy cycle was completed in patients with LCAA who received BD or DBD regimens. The exclusion criteria were: (1) dilated cardiomyopathy; (2) hypertrophic cardiomyopathy; (3) Anderson–Fabry disease; (4) acute myocardial infarction; (5) ischemic cardiomyopathy; (6) malignancy other than multiple myeloma. This study was approved by the Ethics Committee on Scientific Research of Qilu Hospital of Shandong University (No. KYLL‐2018‐321). Before subjects were included in the study, written informed consent in accordance with the Declaration of Helsinki were obtained from each subject. All clinical data were retrieved from the electronic medical record system in each enrolled patient, including medical history, physical examination, laboratory tests, ECG, echocardiograms, bone marrow histology, tissue biopsy results, and medications. All patients were classified according to the Mayo 2004 Staging System with European modification, and the NYHA functional classification. Patients with baseline NT‐proBNP levels > 332 ng/L and baseline cTnT > 0.035 µg/L or cTnI > 0.01 µg/L were classified as Mayo Stage III who were further classified as Mayo Stage IIIa (baseline NT‐proBNP ≤ 8500 ng/L) and Mayo Stage IIIb (baseline NT‐proBNP > 8500 ng/L).

**FIGURE 7:**
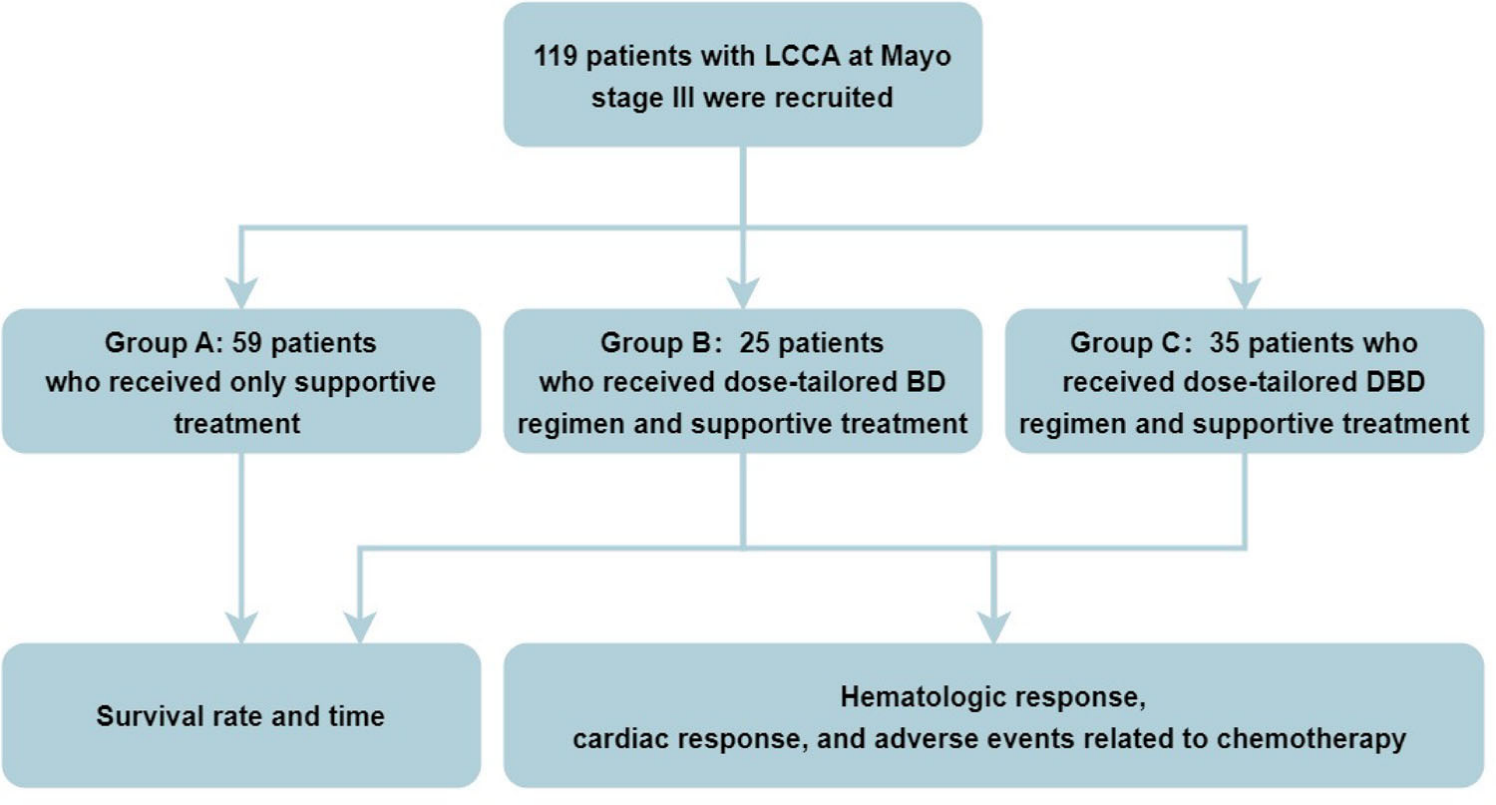
Study flow diagram. BD, bortezomib and dexamethasone; DBD, daratumumab, bortezomib, and dexamethasone.

As shown in Figure 7, based on the therapeutic strategies, all patients were divided into three groups: group A received supportive therapy only including diuretics, beta‐blockers and amiodarone as indicated. Anti‐plasma cell therapy was not used in this group due to denial by these patients for fear of side effects; group B received BD regimen including bortezomib (BSP Pharmaceuticals S.p.A, Italy) at a dose of 1.0 mg/m^2^ injected subcutaneously, and dexamethasone (Chenxin Pharmaceutical Co., Ltd., China) at a dose of 10 mg injected intravenously, and both medications were used at Day 1, 4, 8, and 11 for one chemotherapy cycle; group C received DBD regimen involving daratumumab (Cilag AG, Switzerland) at a dose of 12 mg/kg injected intravenously, bortezomib at a dose of 1.0 mg/m^2^ injected subcutaneously, and dexamethasone at a dose of 10 mg injected intravenously, and the three medications were used weekly for four times as one chemotherapy cycle. Patients receiving daratumumab were premedicated with antipyretics and corticosteroids to prevent drug‐induced fever. The number of chemotherapy cycles depends on hematologic and cardiac responses of treated patients. After two cycles of chemotherapy, patients underwent an evaluation of hematologic response. If there was no evidence of progression, the treatment was continued with another four cycles of chemotherapy. Thereafter, patients were evaluated for hematological and cardiac responses. If there was no hematological or cardiac progression, chemotherapy was discontinued and follow‐up was conducted monthly for 1 year, and thereafter every 3 months. If patients experienced hematological or cardiac progression after six cycles of chemotherapy, BD or DBD treatment together with an immunomodulator was implemented for these patients with refractory LCAA.

The sample size was estimated based on previous statistics, which reported that the 2‐year mortality rate for patients with high‐risk LCCA receiving no chemotherapy was 100% [26]. For patients treated with bortezomib, the 2‐year mortality rate for patients with LCCA was 54% [27]. For patients treated with daratumumab, the 2‐year mortality rate for patients with LCCA was 10% [28]. The significance level (*α*) is set at 0.05 (two‐sided test), and the power of the test (1 − *β*) is 0.80, taking into account a 10% drop‐out rate in a cohort of patients with LCCA followed for more than 2 years. Using PASS software, at least 20 patients in each of the control and treatment groups are needed.

### 5.2 Electrocardiography

Conventional 12‐lead ECG were recorded in all patients and the typical ECG features of LCCA, that is, a low voltage in the limb leads and Q‐waves and poor R‐wave increments in leads V1–V3, were registered.

### 5.3 Echocardiography

Echocardiography was performed in all subjects according to American Society of Echocardiography (ASE) guidelines [29]. Cardiac images were acquired with Vivid E95 GE Ultrasound instrument and stored in DICOM format for offline processing using the EchoPAC (Version 204 GE) workstation. Interventricular septal thickness at end‐diastole (IVSd) and LV posterior wall thickness at end‐diastole (LVPWd) were measured from the parasternal long‐axis view at the level of the mitral valve leaflet tips. LVEF was measured in the apical two‐ and four‐chamber view with a biplane modified Simpson's algorism. Using the 2D strain mode in EchoPAC (Version 204 GE) workstation, the global longitudinal strain (GLS) of the left ventricle was determined.

### 5.4 Laboratory Assays

Blood samples were collected from all patients to determine the blood cell count, hemoglobin (HGB) level, and serum levels of albumin (Alb), alkaline phosphatase (AKP), creatinine (Cr), N‐terminal probrain natriuretic peptide (NT‐proBNP), and cardiac troponin I (cTnI). Based on age, sex, and Cr level, glomerular filtration rate (eGFR) was estimated. Serum protein electrophoresis and blood/urine immunofixation electrophoresis were performed to measure immunoglobulin level using an electrophoretic system (Sebia, France). Serum and urine levels of free light chain (FLC) were determined using protein analyzer Siemens BNII (Siemens, Germany). The difference between abnormal and normal serum‐free light‐chain levels (dFLC) was calculated.

### 5.5 Bone Marrow Histology

Bone marrow aspiration and biopsy (BMAB) were performed in all patients. Under local anesthesia with 2% lidocaine, a volume of 0.2 mL of bone marrow fluid was extracted, and 5–7 peripheral blood smears were promptly prepared, followed by staining of three peripheral blood smears after drying. Additionally, 3–5 mL of bone marrow fluid was collected and stored in heparin‐anticoagulant tubes for subsequent flow cytometry analysis. In LCCA patients, the abnormal plasma cells among nucleated bone marrow cells are featured by CD38+/CD138+. Bone marrow biopsy was conducted to obtain a cylindrical bone marrow tissue with a diameter of 2 mm and a length of 10 mm. Following tissue fixation, immunohistochemical staining and special staining were conducted.

### 5.6 Tissue Biopsy

Tissue biopsy was performed in all recruited patients. Under local anesthesia with 2% lidocaine, tissue samples were taken from myocardium, kidney, liver, abdominal subcutaneous adipose tissue, extremity neuromuscular tissue, tongue, bladder, gastrointestinal tract, or bone marrow, as indicated. The presence of amyloid deposition was confirmed by a positive Congo red staining in a tissue sample. During the staining process, Congo red aqueous solution (Beijing Yili, China) was applied onto the tissue sections, and amyloid deposits in tissue exhibited brick‐red color. When viewed under polarized light, amyloid deposits exhibited apple green birefringence. Diagnosis of LCCA was established after histological identification and accurate typing of amyloid deposits with immunoelectron microscopy.

### 5.7 Therapeutic Efficacy of Chemotherapy

All recruited patients were followed up at the outpatient clinic of Department of Cardiology, Shandong University, and hematologic response was assessed in group B and group C based on immunofixation electrophoresis, serum‐free light chain (sFLC), and dFLC levels. By referring to previously defined criteria [30], homological response to chemotherapy in the present study was classified as: (1) CR: negative finding during blood/urine immunofixation electrophoresis and normal FLC levels and *κ*/*λ* FLC ratios; (2) VGPR: dFLC level is reduced to < 40 mg/L; (3) PR: for patients with baseline dFLC > 50 mg/L, dFLC is decreased by > 50% and for patients with dFLC between 20 and 50 mg/L, dFLC is < 10 mg/L; (4) no response (NR): less effective than PR; (5) progressive disease (PD): defined as the following: in patients with CR, serum M protein is detected again or FLC ratios become abnormal with sFLC levels increased by twofold; or serum M protein level increases by ≥ 50% and greater than 5 g/L; urine M protein increases by > 50% and greater than 200 mg/day; or FLC levels increase by 50% and greater than 100 mg/L; PR or PD criteria are not met; (6) ORR: the sum of patients with CR, VGPR, and PR.

Cardiac response to chemotherapy was evaluated in group B and group C based on serum levels of NT‐proBNP and cTnI levels, and echocardiographic measurement of LVEF. By referring to previously defined criteria [30], cardiac response to chemotherapy in the present study was classified as: (1) CR: NT‐proBNP ≤ 350 ng/L and BNP ≤ 80 ng/L; (2) VGPR: NT‐proBNP level decreases by > 60%; (3) PR: NT‐proBNP level decreases by 31%–60%; (4) NR: NT‐proBNP level decreases by ≤ 30%; (5) PD: NT‐proBNP level increases by > 30% and reaches > 300 ng/L, or cTnI level increases by > 33%, or LVEF decreases by > 10%; (6) ORR: the sum of patients with CR, VGPR, and PR. Furthermore, categorical changes of the NYHA cardiac function class after chemotherapy were analyzed in group B and group C. Finally, survival rate and the median survival time were calculated in three groups of recruited patients.

### 5.8 Adverse Events of Chemotherapy

Throughout the chemotherapy duration, adverse reactions were assessed in accordance with the Common Terminology Criteria for Adverse Events version 5.0 [31] as follows: Grade 1: mild; asymptomatic or with mild symptoms, clinical or diagnostic observations only and intervention not indicated; Grade 2: moderate; minimal, local, or noninvasive intervention indicated, limiting age‐appropriate instrumental activities of daily living, and Grade 3: severe or medically significant but not immediately life‐threatening; hospitalization or prolongation of hospitalization indicated, disabling, limiting self‐care activities of daily living. If a patient experiences life‐threatening consequences or requires urgent treatment, it is assessed as Grade 4 adverse events.

### 5.9 Statistical Analysis

A SPSS 25.0 statistical package program (SPSS, Inc., IL, USA) was used to analyze the data. All measurements were tested for normality using Shapiro–Wilk, and those conforming to a normal distribution were expressed as mean ± standard deviation and analyzed using the one‐way ANOVA test or *t*‐test, while those not conforming to a normal distribution were expressed as median and interquartile spacing and analyzed using the Kruskal–Wallis *H*‐test. Continuous data were expressed as percentages (%) and analyzed with chi‐square test. Time‐to‐event data and the median survival time were assessed using the Kaplan–Meier method (log‐rank test). Univariate and multivariate Cox‐regression analysis was applied to derive prognostic factors. The HRs of various variables with their 95% CI were calculated to assess their effects on outcomes.

## Author Contributions

Yun Ti and Huaitao Yu contributed to acquisition of data, statistical analysis, and interpretation of the results. Ying Wang, Mei Ni, Tongtao Liu, and Luqun Wang contributed to acquisition of data. Yun Zhang, Peili Bu, and Cheng Zhang contributed to project design and administration, writing review and editing, and funding acquisition. All the authors have read and approved the final manuscript.

## Ethics Statement

The investigation conformed with the principles outlined in the Declaration of Helsinki and was approved by the Ethics Committee on Scientific Research of Qilu Hospital of Shandong University (No. KYLL‐2018‐321). Informed consent was obtained from all enrolled subjects in this study.

## Conflicts of Interest

The authors declare no conflicts of interest.

## Supporting information



Supporting Information

## Data Availability

The data of this study are available in the article and online Supporting Information.
