# Supplementary material for: A Dose‐Tailored Anti‐Plasma Cell Regimen Lowers the Mortality of Late‐Stage Cardiac Amyloidosis
Source: MedComm (2020). 2025 Jul 2;6(7):e70219. doi: 10.1002/mco2.70219 (PMC12214942; doi:10.1002/mco2.70219)
Supplement: Supplementary file 1 — Supporting Information [file MCO2-6-e70219-s001.pdf]

Supplementary table 1. Comparison of cardiac response to chemotherapy of 3 months between group B and group C

Characteristics	Group B (n=25)	Group C (n=35)	*P* value
Cardiac response, n (%)			
ORR	6 (24.0%)	13 (37.1%)	0.281
≥VGPR	2 (8.0%)	3 (8.6%)	1.000
VGPR	2 (8.0%)	3 (8.6%)	
PR	4 (16.0%)	10 (28.6%)	
NR	15 (76.0%)	22 (62.8%)	

Abbreviations: CR, complete remission; NR, no response; ORR, overall response rate; PR, partial response; VGPR, very good partial response.

Fig. S1

A

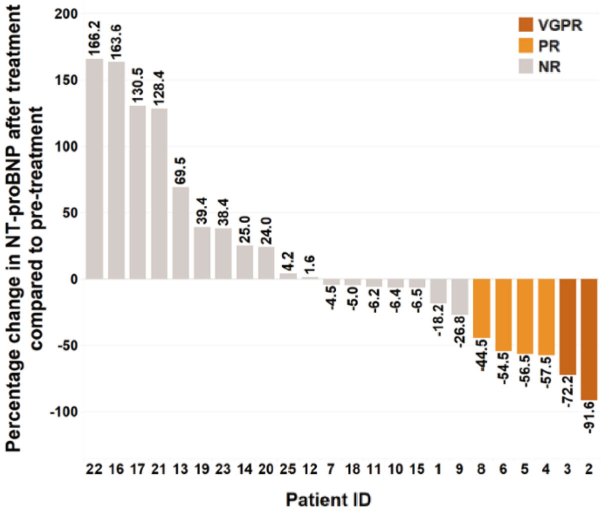

B

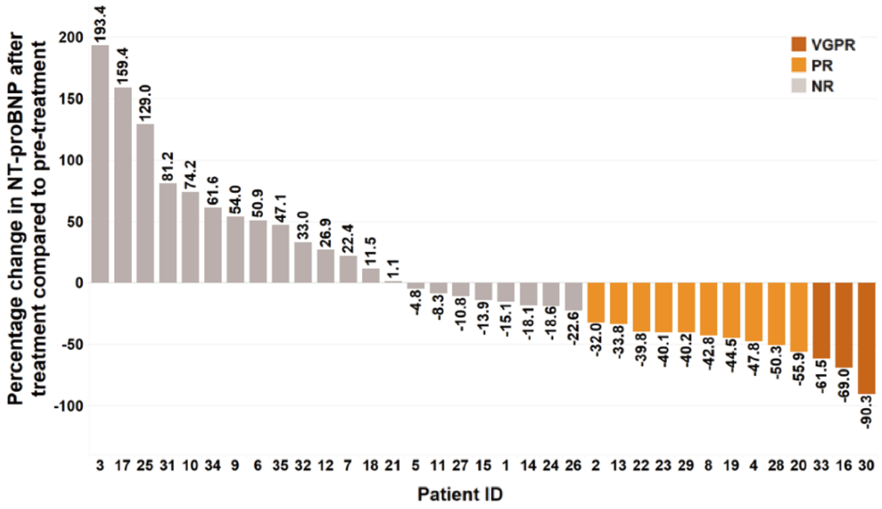

Figure S1. Percent change of NT-proBNP 3 months after the initiation of chemotherapy in group B and group C. Waterfall plot showing percent change of NT-proBNP 3 months after the initiation of chemotherapy in each patient of group B (A) and group C (B). PR, partial response; VGPR, very good partial response; NR, no response; NT-proBNP, N terminal-pro B type natriuretic peptide.

Fig. S2

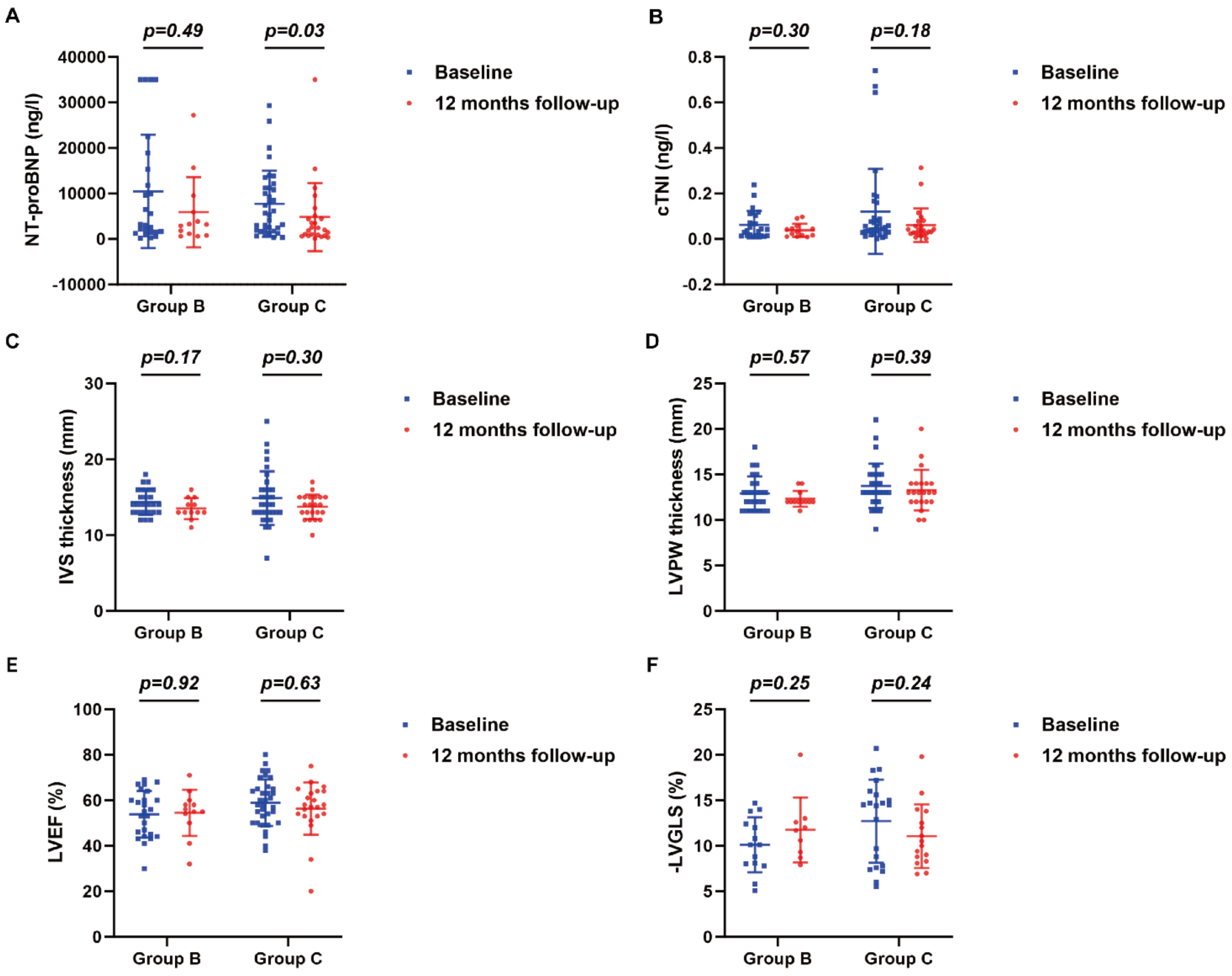

Figure S2. Changes in NT-proBNP, cTnI, and echocardiographic measurements 12 months after the initiation of chemotherapy in group B and group C. Scatter plots representing changes in NT-proBNP (A), cTnI (B), IVS thickness (C), LVPW thickness (D), LVEF (E), LVGLS (F) 12 months after the initiation of chemotherapy in group B and group C. P value: Mann-Whitney U test (Wilcoxon rank sum test) for NT-proBNP, cTnI, IVS thickness, LVPW thickness, and LVEF; Independent samples T-test for LVGLS. Statistical significance: $P < 0.05$. N = 13-35 for NT-proBNP and cTnI, N = 12-33 for IVS thickness and LVPW thickness, and N = 12-35 for LVEF，N = 9-21 for LVGLS. NT-proBNP, N terminal-pro B type natriuretic peptide; cTNI, cardiac troponin I; IVS, interventricular septal; LVPW, left ventricular posterior wall; LVEF, left ventricular ejection fraction; LVGLS, left ventricular global longitudinal strain.
